# A high-efficiency transient expression system mediated by *Agrobacterium tumefaciens* in *Spinacia oleracea* leaves

**DOI:** 10.1186/s13007-024-01218-y

**Published:** 2024-07-02

**Authors:** Yumeng Zhang, Liuliu Qiu, Yongxue Zhang, Yiran Wang, Chunxiang Fu, Shaojun Dai, Meihong Sun

**Affiliations:** 1 Development Center of Plant Germplasm Resources, College of Life Sciences, Shanghai Normal University, Shanghai, 200234 China; 2 Shanghai Key Laboratory of Protected Horticulture Technology, Horticultural Research Institute, Shanghai Academy of Agricultural Science, Shanghai, 201403 China; 3 Shandong Technology Innovation Center of Synthetic Biology, Shandong Provincial Key Laboratory of Energy Genetics and CAS Key Laboratory of Biofuels, Qingdao Institute of Bioenergy and Bioprocess Technology, Chinese Academy of Sciences, Qingdao, 266000 China

**Keywords:** Transient gene expression, Spinach, Subcellular localization, Protein-protein interaction

## Abstract

**Background:**

Optimization of a highly efficient transient expression system is critical for the study of gene function, particularly in those plants in which stable transformation methods are not widely available. *Agrobacterium tumefaciens*‑mediated transient transformation is a simple and low-cost method that has been developed and applied to a wide variety of plant species. However, the transient expression in spinach (*Spinacia oleracea* L.) is still not reported.

**Results:**

We developed a transient expression system in spinach leaves of the Sp75 and Sp73 varieties. Several factors influencing the transformation efficiency were optimized such as *Agrobacterium* strain, spinach seedling stage, leaf position, and the expression time after injection. *Agrobacterium* strain GV3101 (pSoup-p19) was more efficient than AGL1 in expressing recombinant protein in spinach leaves. In general, Sp75 leaves were more suitable than Sp73 leaves, regardless of grow stage. At four-leaf stage, higher intensity and efficiency of transient expression were observed in group 1 (G1) of Sp75 at 53 h after injection (HAI) and in G1 of Sp73 at 64 HAI. At six-leaf stage of Sp75, group 3 (G3) at 72 HAI were the most effective condition for transient expression. Using the optimized expression system, we detected the subcellular localization of a transcriptional co-activator SoMBF1c and a NADPH oxidase SoRbohF. We also detected the interaction of the protein kinase SoCRK10 and the NADPH oxidase SoRbohB.

**Conclusion:**

This study established a method of highly efficient transient expression mediated by *Agrobacterium* in spinach leaves. The transient expression system will facilitate the analysis of gene function and lay a solid foundation for molecular design breeding of spinach.

**Supplementary Information:**

The online version contains supplementary material available at 10.1186/s13007-024-01218-y.

## Introduction

Spinach (*Spinacia oleracea* L., 2 *N* = 2× =12) is an annual or biennial green leafy vegetable that belongs to the Amaranthaceae family [1, 2]. Spinach is an economically important crop due to its high amounts of vitamins, minerals, and antioxidant compounds [3–5]. However, spinach cultivation and production are severely threatened by various stress factors such as pathogens, salinity, drought, and heat [6–8].

A high-quality genome assembly of a Chinese spinach cultivar, Sp75, has been reported [2, 9] and genome sequencing of 305 cultivated and wild spinach accessions was performed [10]. Importantly, transcriptome sequences from 120 cultivated and wild spinach accessions and the genome-wide association studies (GWAS) of important spinach agronomic traits provided candidate genes for further functional analysis [2, 10]. Furthermore, RNA-Seq expression profiling and proteomic analyses revealed hundreds of heat-responsive genes and proteins in spinach, implicating transcriptional, translational, and post-translational regulation in response to heat stress [7, 8, 11]. However, the molecular regulatory mechanisms are still unknown due to the lack of a highly -efficient genetic transformation system in spinach.

The transient expression technique allows rapid introduction of target genes into plant organisms through plant-specific expression vectors, leading to their expression in a short period of time (up to several days) [12]. The transient expression system can overcome some limitations inherent to the stable genetic transformation system such as the low regeneration efficiency and complicated tissue culture procedures [13]. Therefore, it has become a rapid, convenient and effective method and has been widely applied in the analysis of promoter activity, protein-protein interaction, and gene function, as well as the high-throughput screening [14–17].

There are several transient expression methods widely used in plants, which depend on different media, such as *Agrobacterium* infiltration, gene gun, polyethylene glycol (PEG), and plant viral vector [18–21]. Among them, the *Agrobacterium* infiltration-mediated transient expression system has the advantages of simple and efficient operation, which has been applied in different tissues/organs of various plants such as leaves of tobacco (*Nicotiana benthamiana*) [22], strawberry (*Fragaria* × *ananassa* Duch.) [23], citrus (*Fortunella margarita*) [13], *Brachypodium distachyon* [24], sunflower (*Helianthus annuus*) [25] and medicinal plant [26], tomato fruits (*Solanum lycopersicum*) [27], apple calluses (*Malus domestica*) [28], as well as lily petals (*Lilium brownii* var. *viridulum Baker*) [29]. Furthemore, the technique of transient expression mediated by *Agrobacterium* infiltration has also been successfully applied for the production of commercial recombinant proteins (e.g., plantibodies, vaccines, and therapeutics) [30, 31]. However, there are no reports on spinach.

The multiprotein bridging factor 1 (MBF1) proteins function as transcriptional co-activators, which bridge transcription factors and the basal transcription machinery [32]. In plants, MBF1 proteins have been demonstrated to be involved in specific developmental processes and differential stress responses [32]. SoMBF1c is a member of the SoMBF1 family in spinach [33]. NADPH oxidases (NOXs), also named respiratory burst oxidase homologues (Rbohs), are essential producers of reactive oxygen species (ROS) at the plasma membrane [34, 35]. The *Arabidopsis* genome contains 10 Rboh genes (AtRbohA-J) [34, 35]. AtRbohD was shown to form a complex with and to be phosphorylated by protein kinase CYSTEINE-RICH RLK2 (CRK2) in response to bacterial pathogen [36]. Cysteine-rich receptor-like kinases (CRKs) represent one of the largest subfamilies of receptor-like kinases (RLKs), which are plasma membrane-bound receptors that are ubiquitous in higher plants [37]. The homologous genes of AtRbohD and AtRbohF in spinach, designated SoRbohB and SoRbohF, respectively, were identified based on the amino acid sequence. SoCRK10 was identified as a member of the SoCRKs family in spinach.

In the present study, a simple and efficient, *Agrobacterium*-mediated spinach leaf transformation system was developed. By comparing fluorescence intensity and transformation efficiency, we investigated the factors that influence and determine the optimal experimental condition, including *Agrobacterium* strain, spinach variety, seedling stage, leaf position, and expression time after injection. Furthermore, this transient transformation system was applied to study the protein localization of SoMBF1c-GFP and SoRbohF-GFP as well as the protein-proteins interaction between SoCRK10 and SoRbohB in spinach.

## Results

### Stages of *Agrobacterium-* mediated transient transformation of spinach leaves

**Fig. 1:**
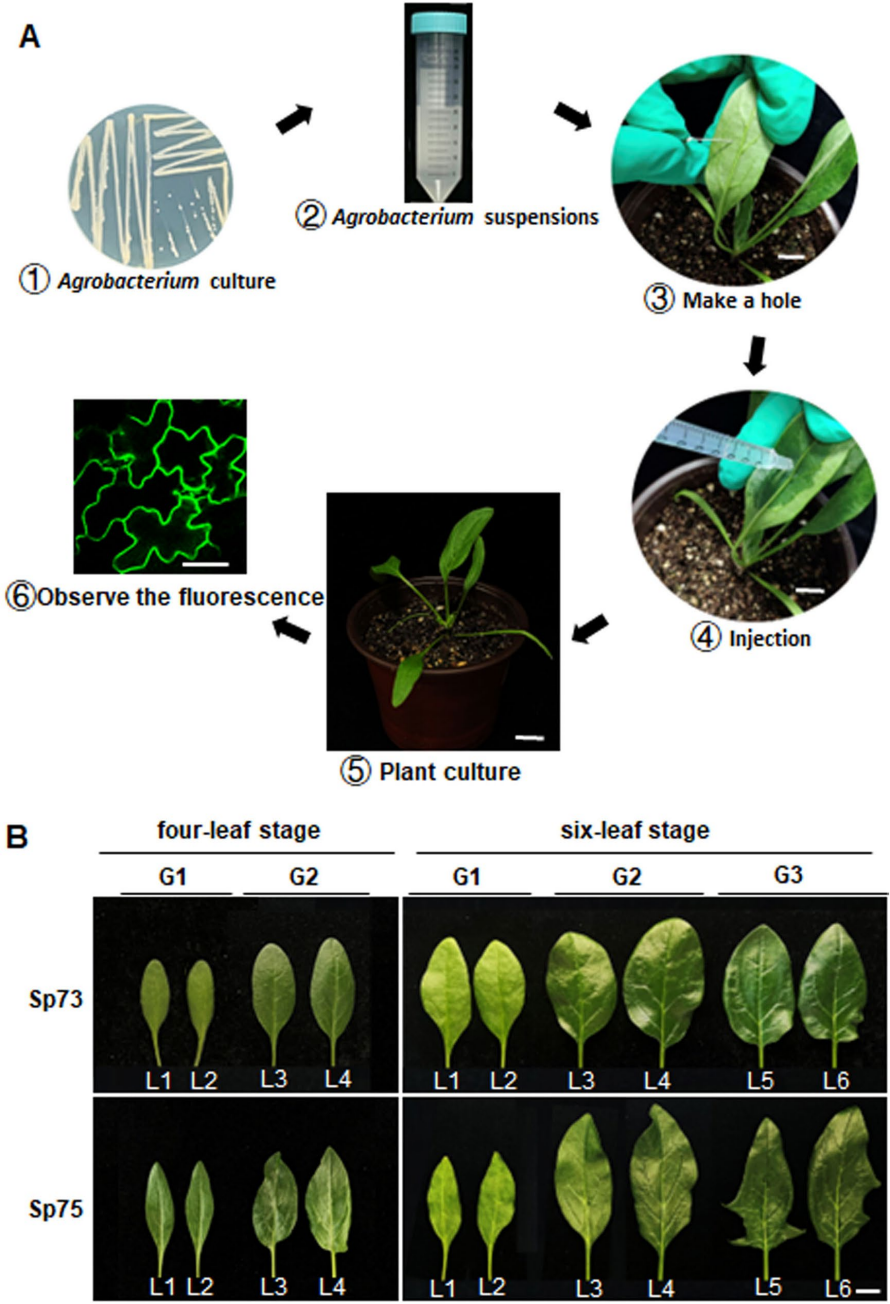
*Agrobacterium* -mediated transient expression procedure in spinach leaves of Sp75 and Sp73. A. Schematic representation of *Agrobacterium*-mediated transient expression procedure: *Agrobacterium* was streaked on LB plate and cultured for 48–72 h (①), then diluted with infiltration medium to obtain *Agrobacterium* suspensions (②); A hole was made with a needle on the abaxial side of spinach leaf (③) and *Agrobacterium* suspensions were injected into the leaves (④); The transformed spinach plants were cultured in darkness for 2 ∼ 3 days (⑤) to observe the fluorescence signal using confocal microscope (⑥). ③-⑤: bar = 1 cm; ⑥: bar = 50 μm. B. A representative image of Sp75 and Sp73 leaves at four-leaf stage (left) and six-leaf stage (right) used for injection. G 1, the first group including the first leaf (L1) and second leaf (L2); G 2, the second group including the third leaf (L3) and fourth leaf (L4); G 3, the third group including the fifth leaf (L5) and sixth leaf (L6). Scale bar = 1 cm

The infiltration of *Agrobacterium* cells into spinach leaves was performed according to the agroinfiltration procedure (Fig. 1A) and the experimental conditions were optimized. Appropriate selection of leaves for *Agrobacterium* agroinfiltration is critical for the success of transient gene expression [38]. In this case, two varieties were used, Sp75, a heat-resistant spinach variety with fleshy leaves, and Sp73, a heat-sensitive variety with waxy leaves. 1 mL syringe was used to perform the infiltration with *Agrobacterium.* [8]. To determine the state and appropriate position of the leaves of spinach seedlings for agroinfiltration, these were categorised into three groups: the first group (G1) was formed by the first (L1) and second (L2) leaves, the second group (G2) was formed by the third (L3) and fourth (L4) leaves, and the third group (G3) was formed by the fifth (L5) and the sixth (L6) leaves (Fig. 1B). The efficiency of transient expression in spinach leaves of Sp75 and Sp73 at the four-leaf and six-leaf stages, respectively, was then evaluated. The efficiency of transient expression was assessed by recording the fluorescence signal of the HopF2-GFP fusion protein, which served as a plasma membrane marker [39].

### Experimental evaluation conditions in the four-leaf stage of Sp75 and Sp73

**Fig. 2:**
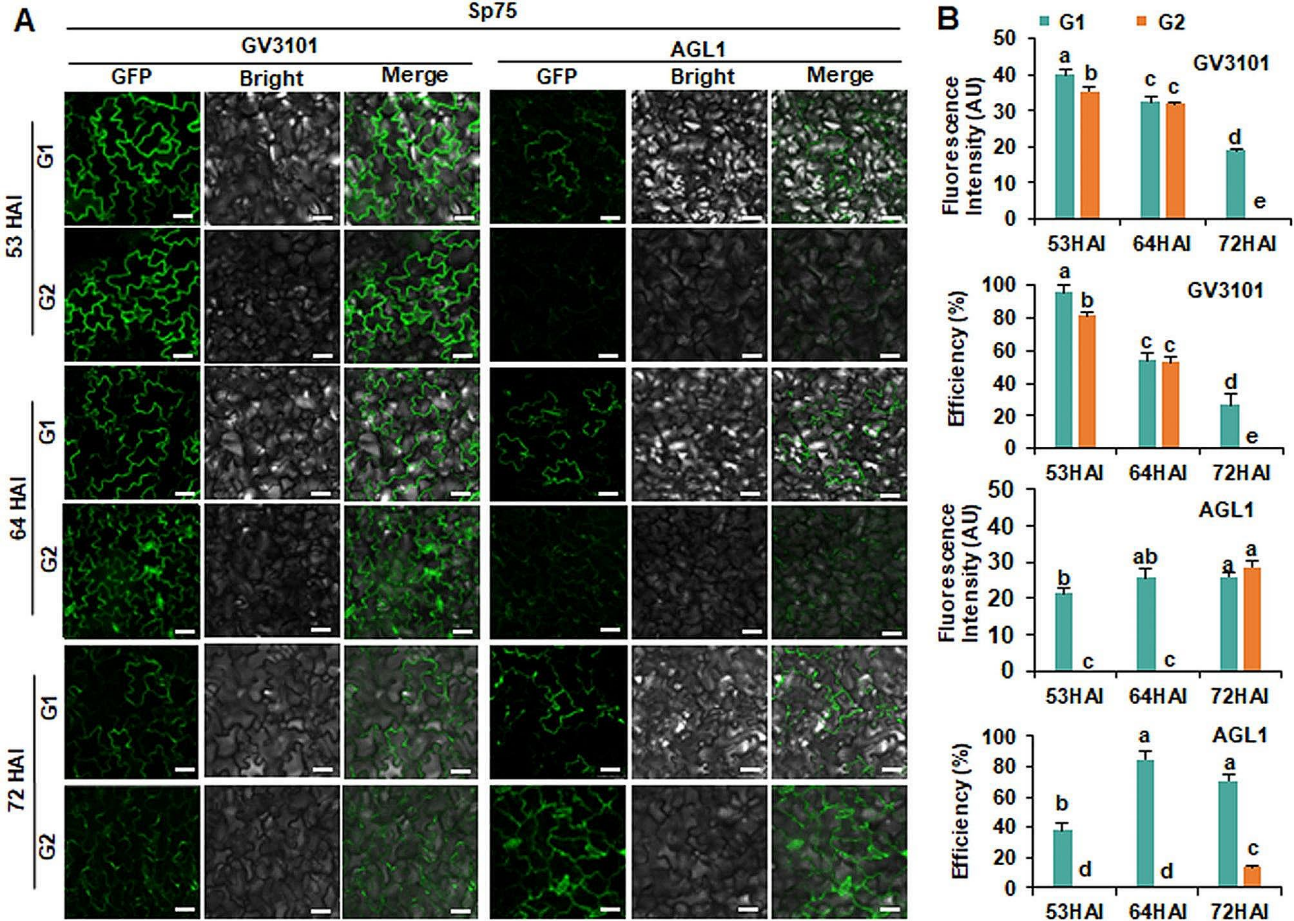
Transient expression of HopF2-GFP in spinach Sp75 at the four-leaf stage. (**A**) GFP fluorescence images. G1 and G2 were infiltrated with *Agrobacterium* strain GV3101 (pSoup-p19) and AGL1 harboring pCAMBIA2300-HopF2-GFP constructs to observe the GFP fluorescence signal at 53 h, 64 h, 72 h after injection (HAI). Scale bar = 50 μm. (**B**) Fluorescent intensity and transformation efficiency of HopF2-GFP in Sp75 leaves using *Agrobacterium* strain GV3101 (pSoup-p19) and AGL1 at 53HAI, 64 HAI, 72HAI. AU: arbitrary unit. The experiments were repeated three times with similar results. Data presented are mean values ± SD (*n* ≥ 6). Different letters represent significant differences determined by one-way ANOVA with Tukey’s post hoc test (*P* < 0.05)

Previous research indicates that several factors, such as *Agrobacterium* strains, plant age and expression time after infiltration, can affect the level of transient gene expression in plant cells [40]. Two *Agrobacterium* strains, GV3101 (pSoup-p19) and AGL1, were used for the infiltration of spinach leaves in order to determine the efficiency of each one. These strains, carrying the plant expression vector pCAMBIA2300-HopF2-GFP, were separately infiltrated into G1 and G2 of Sp75 to determine the most suitable leaves (Fig. 2A). For *Agrobacterium* strain GV3101 (pSoup-p19), the fluorescence intensity and transformation efficiency of HopF2-GFP decreased with increasing expression time from 53 h after infiltration (HAI) to 72 HAI, either in G1 or G2 (Fig. 2B). This is consistent with the expression of HopF2-GFP, which was quantified by qRT-PCR and Western blot assay (Supplementary Figure S1). At 53HAI, HopF2-GFP was greater in G1 than in G2 (Fig. 2B; Supplementary Fig. S1). G1 showed the highest fluorescence intensity (40 AU) and transformation efficiency (95.45%). When the expression time reached 72 h, only very weak fluorescence (19AU) was observed in G1 with low transformation efficiency (26.67%) (Fig. 2A and B; Supplementary Fig. S1), while no GFP signal was detected at 72 HAI of G2 (Fig. 2A and B). In the case of AGL1, HopF2-GFP was successfully expressed in G1 at all three observations times, with the highest efficiency (85%) at 64 HAI (Fig. 2B). However, HopF2-GFP was only detectable in G2 at 72 HAI with a very low efficiency (13.39%). GV3101 (pSoup-p19), compared to AGL1, was more efficient in the transient expression of the gene in Sp75 leaves (Fig. 2).

**Fig. 3:**
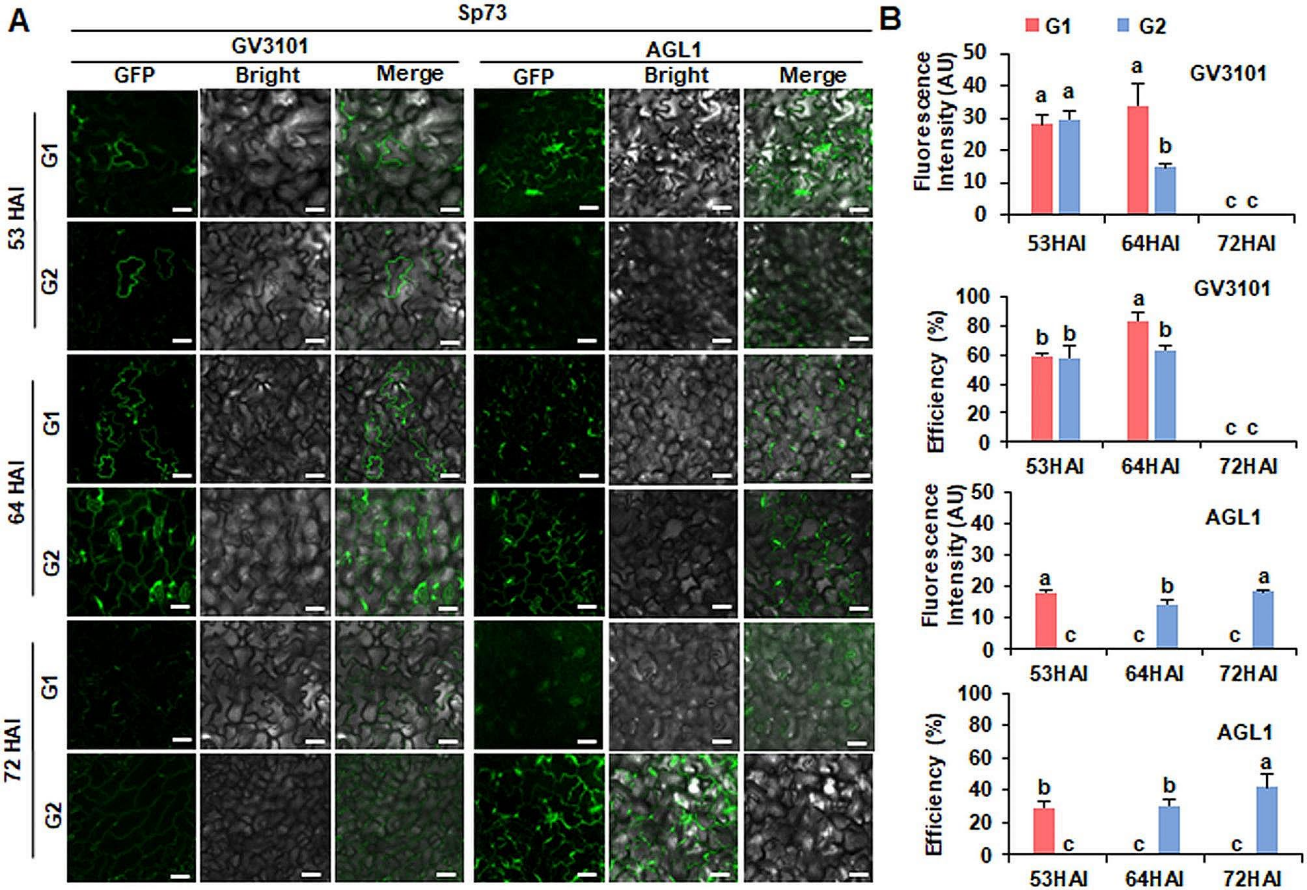
Transient expression of HopF2-GFP in spinach Sp73 at the four-leaf stage. (**A**) GFP fluorescence images. G1 and G2 were infiltrated with *Agrobacterium* strain GV3101 (pSoup-p19) and AGL1 harboring pCambia2300-HopF2-GFP constructs to observe fluorescence signal at 53 h, 64 h, 72 h after injection (HAI). Scale bar = 50 μm. (**B**) Fluorescent intensity and transformation efficiency of HopF2-GFP in Sp73 leaves using *Agrobacterium* strain GV3101 (pSoup-p19) and AGL1 at 53HAI, 64 HAI, 72HAI. AU: arbitrary unit. The experiment was repeated three times with similar results. Data presented are mean values ± SD (*n* ≥ 6). Different letters represent significant differences determined by one-way ANOVA with Tukey’s post hoc test (*P* < 0.05)

In the case of Sp73, when infiltrated with GV3101 (pSoup-p19), HopF2-GFP expression was only observed at 53 HAI and 64 HAI, but not at 72HAI (Fig. 3A). At 53 HAI, no significant differences in fluorescence intensity and expression efficiency were observed between G1 and G2 (27.98 AU and 59.33%; 29.72 AU and 58.33%, respectively). At 64 HAI, G1 showed significantly higher fluorescence intensity and efficiency than G2. When infiltrated with AGL1, GFP signal was observed at 53 HAI of G1 (29.17% transformation efficiency), 64 HAI (29.17% transformation efficiency) of G2 and 72HAI of G2 (41.67%) transformation efficiency (Fig. 3B). Importantly, both fluorescence intensity and efficiency with AGL1 were lower than those observed using GV3101 (pSoup-p19) (Fig. 3B). The expression of HopF2-GFP, mediated by GV3101 (pSoup-p19), was significantly higher than that of AGL1 in both Sp75 and Sp73. This was confirmed by qRT-PCR and western blot analysis (Supplementary Fig. S1).

These results showed that the most effective experimental condition for Sp75 and Sp73 at the four-leaf stage was achieved by infiltrating G1 with GV3101 (pSoup-p19). The ideal period for expression of GV3101 (pSoup-p19) is 64 h. Subsequently, GV3101 (pSoup-p19) was used for subsequent studies.

### Optimal experimental conditions for Sp75 and Sp73 leaves at the six-leaf stage

**Fig. 4:**
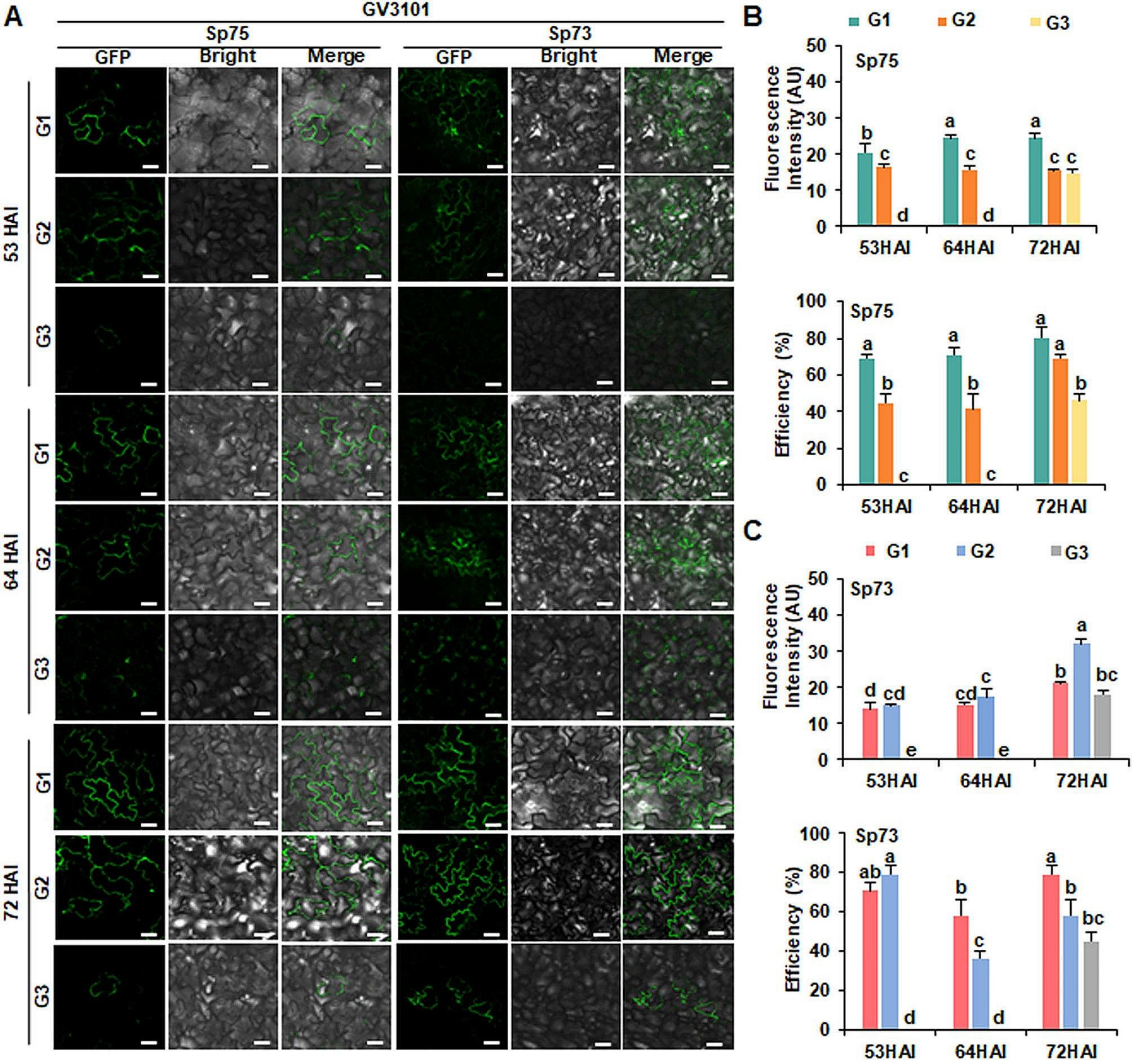
Transient expression of HopF2-GFP in spinach Sp75 and Sp73 at the six-leaf stage. (**A**) GFP fluorescence images. G1, G2 and G3 was infiltrated with *Agrobacterium* strain GV3101 (pSoup-p19) harboring pCambia2300-HopF2-GFP constructs to observe the fluorescence signal at 53 h, 64 h, 72 h after injection (HAI). Scale bar = 50 μm. (**B**) Fluorescent intensity and transformation efficiency of HopF2-GFP in Sp75 and Sp73 leaves using *Agrobacterium* strain GV3101 (pSoup-p19) at 53HAI, 64 HAI, 72HAI. AU: arbitrary unit. The experiment was repeated three times with similar results. Data presented are mean values ± SD (*n* ≥ 6). Different letters represent significant differences determined by one-way ANOVA with Tukey’s post hoc test (*P* < 0.05)

GV3101 (pSoup-p19) carrying the pCAMBIA2300-HopF2-GFP vector was infiltrated into G1, G2 and G3 at the six-leaf stage of Sp75 and Sp73 to observe GFP signal (Fig. 4). The fluorescence imaging results showed the detection of HopF2-GFP in Sp75 and Sp73 at 53 HAI, 64 HAI and 72 HAI in both G1 and G2, while in G3, it was only detected at 72 HAI (Fig. 4A). For Sp75 at 53 HAI, 64 HAI, or 72 HAI, the fluorescence intensity and efficiency of G1 were higher than those of G2 and G3, respectively (Fig. 4B). Furthermore, the fluorescence intensity of G1 at 64 HAI and 72 HAI (23.63 AU and 23.64 AU) was higher than that of G1 at 53 HAI (20.41 AU), confirming that G1 with longer expression time (64 h ∼ 72 h) is more suitable for Sp75 infiltration at the six-leaf stage (Fig. 4B). The expression of HopF2-GFP in G1 of Sp75 at six-leaf stage was decreased compared with that in G1 of Sp75 at four-leaf stage (Supplementary Fig. S1). For Sp73, G2 showed higher fluorescence intensity than G1 and G3 at 53 HAI, 63 HAI, or 72 HAI, and G2 showing the highest fluorescence intensity at 72 HAI. The highest efficiency was achieved by G1 at 72 HAI and G2 at 53 HAI (Fig. 4C). Considering the impact of these two aspects, both G1 and G2 of Sp73 could be used for transient expression at the six-leaf stage (Fig. 4C). Only in terms of fluorescence intensity, G1 of Sp75 at 53 HAI and 64 HAI (20.41 AU and 24.63 AU) was larger than that of Sp73 (14.12 AU and 15.38 AU), while G2 of Sp75 at 72 HAI (15.51 AU) was lower than G2 of Sp73 at 72 HAI (32.17 AU) (Fig. 4B and C). These comparisons indicated that G1 of Sp75 were more effective in the early expression time (53 h ∼ 64 h), while G2 of Sp73 was more effective at long expression time (72 h).

### GV3101(pSoup-p19)-mediated transient expression for analyzing subcellular localization of SoMBF1c and SoRbohF

**Fig. 5:**
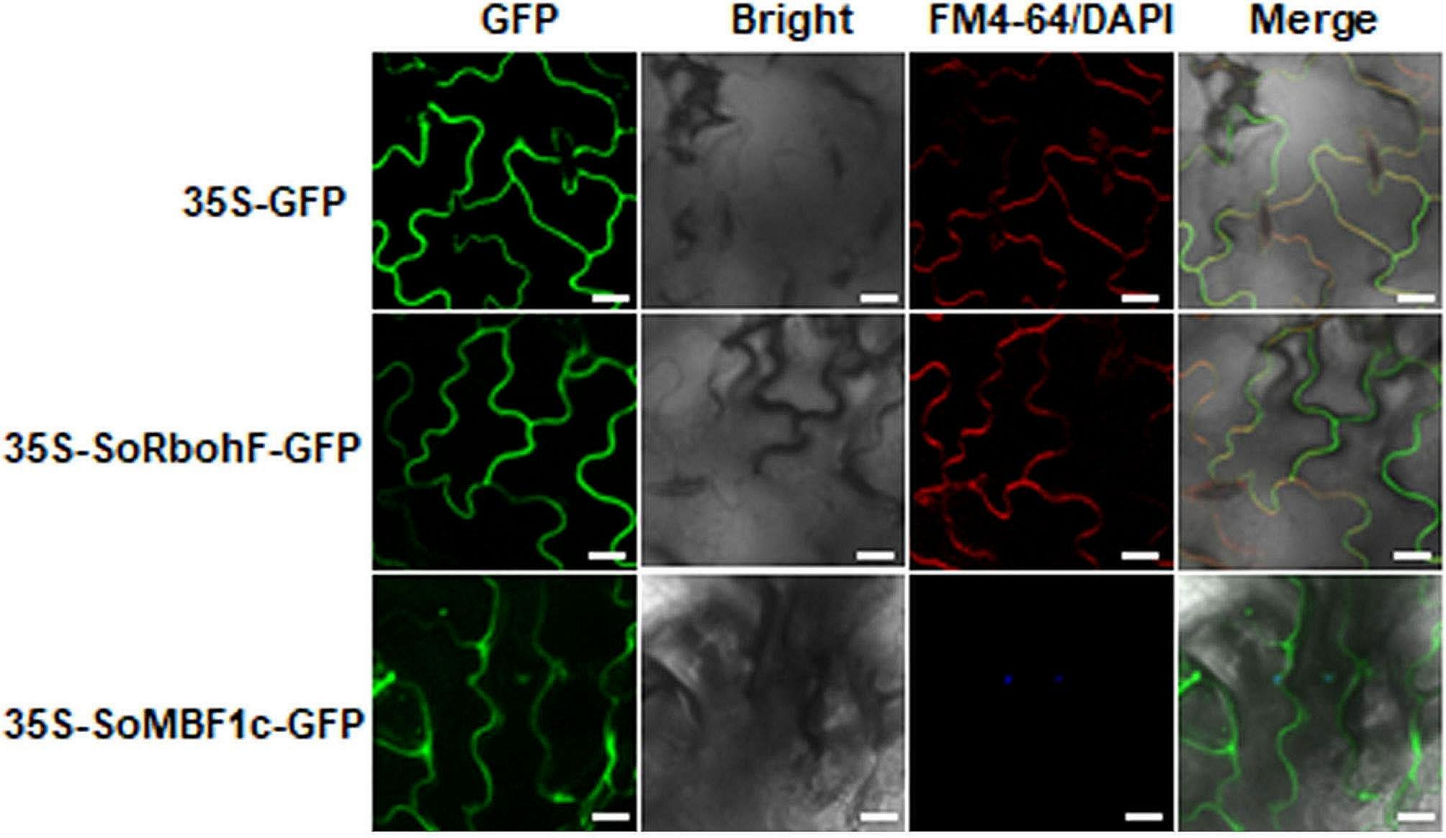
Subcellular localization of SoRbohF and SoMBF1c. The constructs (SoRbohF-GFP, SoMBF1c-GFP or GFP control) were driven by the *Cauliflower mosaic* virus *35 S* promoter and transiently expressed in G1 of spinach Sp75. The GFP and SoMBF1c-GFP was observed by confocal microscopy at 53 h after injection, SoRbohF-GFP was observed at 64 h after injection. Before imaging, spinach leaves were injected with FM4-64 or DAPI for 10 min in dark. Scale bar = 50 μm

MBF1c is a member of the MBF1 family and acts as transcriptional co-activator to regulate gene expression in response to heat stress [33]. The NADPH oxidase RbohF is a plasma membrane localization protein and is required for stomatal immunity by modulating both reactive oxygen species and apoplastic pH dynamics [41, 42]. Subsequently, the subcellular localization of SoMBF1c-GFP and SoRbohF-GFP was analized by GV3101(pSoup-p19)-mediated transient expression in G1 of spinach Sp75 and confocal images were taken at 53 HAI and 64 HAI. The subcellular localization assay revealed that SoMBF1c is a protein that is localized in both the nucleus and cytoplasm (Fig. 5; Supplementary Fig. S2). Moreover, SoMBF1c was found to be co-localized with the nuclear dye DAPI in the nucleus. (Fig. 5). SoRbohF-GFP was observed at the plasma membrane, which co- localized with the cytoplasmic membrane dye FM4-64 (Fig. 5; Supplementary Fig. S2). The fluorescence intensity of SoRbohF-GFP, SoMBF1c-GFP, and the GFP control were similar. This indicates that the transient expression system allows for sufficient protein levels to be expressed in the subcellular localization assay (Supplementary Fig. S2).

### GV3101(pSoup-p19)-mediated transient expression to detect interactions between SoCRK10 and SoRbohB

**Fig. 6:**
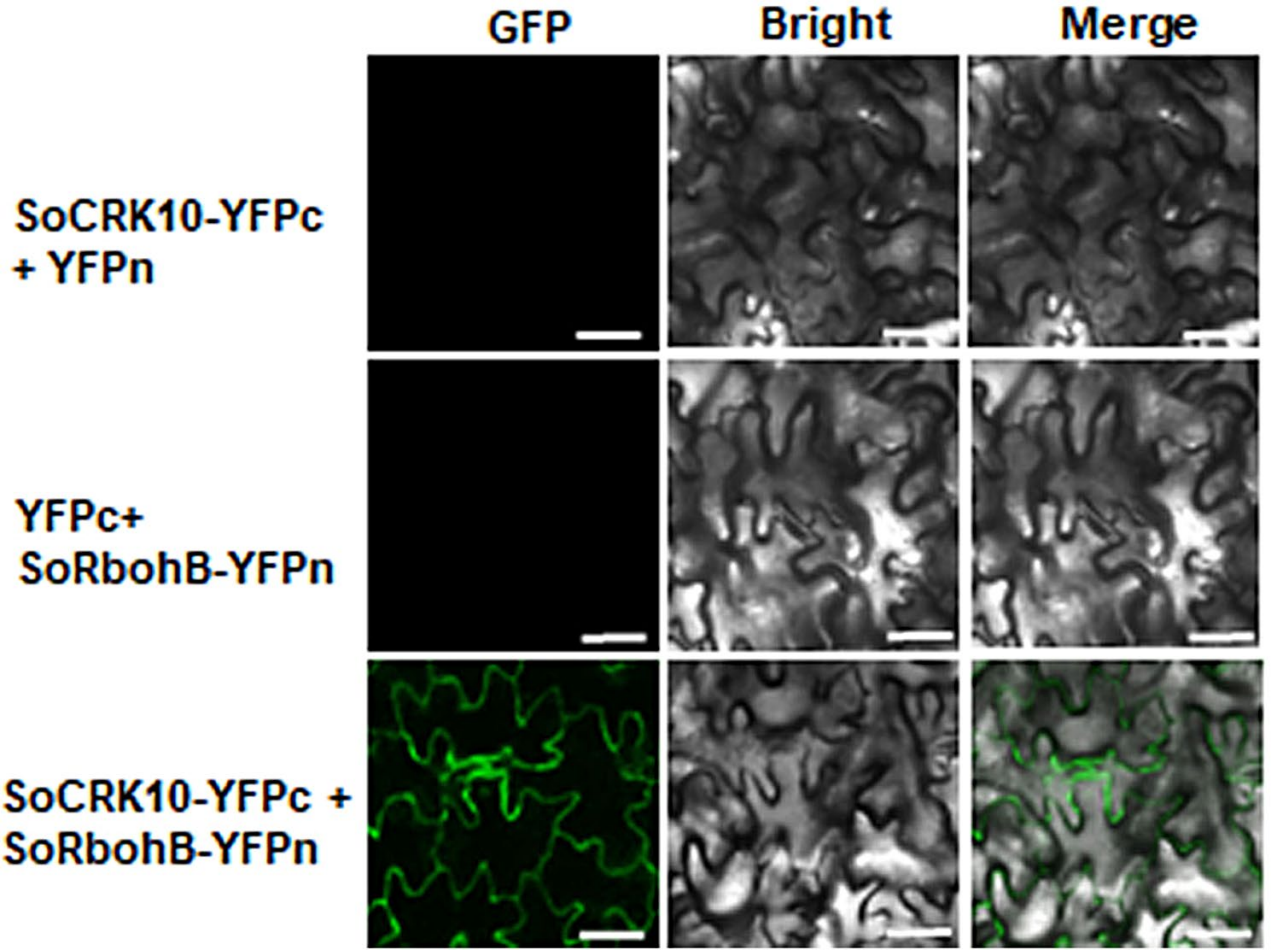
Detection of the interaction between SoCRK10 and SoRbohB by BiFC assays in spinach Sp75 leaves. SoCRK10-YFPc + YFPn and SoRbohB-YFPn + YFPc were used as negative controls. Scale bar = 50 μm

Cysteine-rich receptor-like protein kinases (CRKs) mediated ROS production by interacting with RbohD/F in plant immune responses [37]. Subsequently, the interaction of homologous proteins of SoCRK10 and SoRbohB was confirmed by BiFC assay in Sp75 leaves. No fluorescence signals were observed in control leaves that co-expressed pCAMBIA1300-SoRbohB-YFPn with pCAMBIA1300-YFPc or pCAMBIA1300-SoCRK10-YFPc with pCAMBIA1300-YFPn (Fig. 6). When G1 of Sp75 was co-infiltrated with GV3101 (pSoup-p19) strains carrying the construction pCAMBIA1300-SoCRK10-YFPc and pCAMBIA1300-SoRbohB-YFPn, prominent YFP signals were observed in the injected leaves at 69 HAI (Fig. 6). The BiFC assay showed that SoCRK10 interacted with SoRbohB in vivo. These results indicated that the GV3101(pSoup-p19)-mediated transient transformation method using spinach leaves can provide sufficient recombinant protein of interest to perform protein localization and BiFC assay.

### GV3101(pSoup-p19)-mediated transient expression to perform β-glucuronidase (GUS) assay

The *GUS* reporter gene system is applicable for characterizing gene or protein expression patterns. In order to determine if the spinach leaf transient system is suitable for the GUS tag, we transformed the pCAMBIA1305 vector, which contains the *GUS* gene driven by the *Cauliflower mosaic* virus (CaMV) *35 S* promoter, into the G1 of Sp75 leaves. The spinach leaves infiltrated with GV3101 (pSoup-p19) containing the 35 S::GUS vector exhibited clear blue areas, while no blue signals were detected in the control leaves transformed with GV3101 (pSoup-p19) without vector (Supplementary Fig. S3). The *GUS* reporter gene was successfully expressed in spinach leaves through this *Agrobacterium*-mediated transient system.

## Discussion

*Agrobacterium* strains have become a powerful experimental tool for genetic transformation assays in a wide range of plant species [43]. The capacity for *Agrobacterium*-mediated genetic transformation was determined by the tumor-inducing (Ti) plasmid, which contains essential regions necessary for DNA transfer to the host cell [12]. Ach5 and C58 serve as popular chromosome backgrounds in plant biotechnology and have been optimized over the years to boost transformation efficiency in various plant species. This has been achieved by mutations or by inserting the original Ti plasmid [44, 45]. Commonly used strains, including GV3101, EHA101, EHA105, and AGL1, are derived from C58, while LBA4213 and LBA4404 share the same chromosomal background as Ach5 [45].

Different *Agrobacterium* strains (e.g., LBA4404, AGL1, GV3101, EHA105, and C58C1) have variations in the efficiency of transient gene expression. It is crucial to detect and identify the optimal *Agrobacterium* strain for the target plant to establish the transient expression system and improve the transformation efficiency. Among them, the LBA4404 showed the highest efficiency in mediating transient expression in *Arabidopsis*, as determined by fluorometric measurements of GUS activity and GUS staining [40]. EHA105 was the superior strain in generating GFP signals and with higher LUC activity compared to LBA4404 and GV3101 when the transient expression system was used in the leaves of *Artemisia annua* [26]. GV3101 had the highest levels of transient gene expression in citrus and *Caragana intermedia* leaves through *Agrobacterium*-mediated transformation [46, 47]. GV3101 (pSoup-p19) was found to mediated significantly higher gene expression than AGL1 in spinach leaves, as indicated by higher fluorescence intensity and higher transformation efficiency (Figs. 2 and 3).

Different cultivars frequently present differing sensitivities to *Agrobacterium* infection, highlighting the genetic background’s essential role in *Agrobacterium*-mediated transient expression [48]. Among ten diverse ecotypes of *Arabidopsis* accessions, WS (Wassilewskija) exhibited more significant agroinfiltration capacity than Columbia (Col-0) [38]. Spinach leaves are usually large, flat and smooth, making them very suited for infiltration with *Agrobacterium*. However, leaf characteristics tend to vary in different spinach cultivars. This study revealed that leaves of Sp75were more susceptible to infiltration compared to those of Sp73 (Figs. 2, 3 and 4), possibly due to differences in leaf structure of the two cultivars. Succulent leaves with spacious intercellular spaces of Sp75 helped the penetration of *Agrobacterium* suspension through the syringe infiltration method [38].

The level of transient expression varied according to the position and the age of the leaf for the assay. Younger leaves showed higher transformation efficiency than older ones. The first pair of leaves was used in lettuce (*Lactuca sativa* Linn.), tobacco, tomato and *A. annua* for infiltration with *Agrobacterium* to obtain higher levels of gene expression [26, 38]. Furthermore, the development stage also had an impact on the transient expression levels [49]. It was also confirmed that G1 showed the highest fluorescence intensity, and the four-leaf stage was more suitable than that at the six-leaf stage (Figs. 2, 3 and 4) for transient expression.

The abundance and duration of protein accumulation is different in the various transient expression systems and plant species [50]. In young *Arabidopsis* seeds and using a rapid *Agrobacterium*-mediated Seedling Transformation (FAST) methods, the expression of the marker gene *Renilla reniformis luciferase* (*hRLuc*) reached the maximum level at 36 h of co- culture [51]. *Agrobacterium*-mediated enhanced seedling transformation (AGROBEST) using the *Arabidopsis* immune receptor mutant *efr-1* demonstrated that GUS signals were undetectable at 1 day post infection (dpi) but gradually increased to a plateau at 3 or 4 dpi [52]. Here, we detected the fluorescence intensity at 53 HAI, 64 HAI and 72 HAI with the optimal time of GV3101 (pSoup-p19) in Sp75 being between 53 h and 64 h and should not exceed 72 h. Therefore, it is advisable to perform accumulation analysis for each new protein to confirm the optimal expression time. In addition to the factors evaluated, several molecular factors need to be considered in the future, including the manipulation of agents that promote T-DNA transfer and the use of silencing suppressors [53].

Transient gene expression obtained in plant is sufficient for RNA and protein analysis, with potential for laboratory-scale protein production in a short period of time [54, 55]. Tobacco is the most widely used species to study the gene function using the transient expression method [56, 57]. However, expression of heterologous gene has limited applications for validating gene function. This established and optimized *Agrobacterium*-mediated transient transformation system of spinach leaves will allow more precise analysis of the function of the target gene in homologous plants.

MBF1c, an evolutionarily conserved transcriptional co-activator belonging to the MBF1 gene family, serves as a key regulator of abiotic stress response and plant development [32, 33]. In silkworm (*Bombyx mori*), BmMBF1 was localized in the cytoplasm, but translocated to nucleus at D3 stage of the development [58]. The *Arabidopsis* genome contains three MBF1 genes (*MBF1a*, *MBF1b* and *MBF1c*), which were localized both in the cytoplasm and in nucleus [59]. Plant NOXs genes, also known as Rbohs, are responsible for the production of ROS and play an indispensable role in a wide range of cellular and developmental processes [34]. All Rboh proteins are localized to the plasma membrane and contain transmembrane domains in the C-terminal region [35]. Using this transient method in spinach, nuclear and cytoplasmic localization of SoMBF1c and plasma membrane localization of SoRbohF were observed (Fig. 5). CRKs belong to the RLK family and play key roles in plant immunity, abiotic stress response, and growth and development [37]. In plant immune responses, CRK2 interacted with and phosphorylated RbohD to regulate ROS production [36]. Here we detected the protein-protein interactions of SoCRK10 and SoRbohB through BiFC assay, further demonstrating the conserved CRK-Rbohs molecular regulation module in spinach (Fig. 6). Furthermore, this method can be easily adopted for other types of functional assays such as RNAi and genome editing to studying various regulatory and defense mechanisms in the future [60–62].

## Conclusions

In this study, a transient expression method was established using spinach leaves and different physiological factors affecting the transformation efficiency and gene expression level were evaluated. The applicability to detect the subcellular localization of SoMBF1c and SoRbohF, as well as the protein-protein interactions between SoCRK10 and SoRbohB through BiFC, suggested that this transient expression method is a feasible method to routinely use for functional studies in spinach.

## Materials and methods

### Plant material and growth condition

Two spinach varieties, heat-sensitive Sp73 and heat-resistant Sp75, were collected from the Germplasm Resource Center of Shanghai Normal University. Seeds were placed on wet filter paper saturated with sterile water and stratified in darkness for one week at 4 °C. The sprouting seeds were potted in mixed soil (perlite: vermiculite: peat = 1:2:2, v/v/v) and grown in a growth chamber at approximately 50% relative humidity with 10 h light/ 14 h dark and 22 °C (light) /20°C (dark) cycle. Seedlings grew to the four-leaf stage after ∼ 28 days and to the six-leaf stage after ∼ 35 days, which were used for the transformation assay.

### RNA extraction, gene cloning and plasmids construction

Total RNA was extracted from 35 days old spinach plants using TRIzol reagent (Invitrogen, USA) and the first single-stranded cDNA was obtained by retrotranscription, according to the manufacturer’s instructions for PrimeScript RT Enzyme (TaKaRa, Japan). Full-length coding sequences were amplified by PCR from a spinach cDNA library using High-Fidelity DNA Polymerase (Vazyme, China). For examination of subcellular localization of SoMBF1c and SoRbohF proteins, the amplified products were inserted into the *XbaI* and *SmaI* sites of the binary vector pCAMBIA2300-GFP with Seamless Assembly Mix (ABclonal, USA), respectively, and then generated recombinant constructs SoMBF1c-GFP and SoRbohF-GFP. HopF2 is an effector protein from *Pseudomonas syringae* tomato DC3000 that localizes to the plasma membrane [63]. The pCAMBIA2300-HopF2-GFP construct was provided by Xiangzong Meng (Shanghai Normal University, China). To verify the interaction between SoCRK10 and SoRbohB, the full-length coding sequences of SoCRK10 and SoRbohB were amplified and placed in plasmids pCAMBIA1300-YFPc and pCAMBIA1300-YFPn driven by the *Cauliflower mosaic virus* 35 S promoter, respectively. The vector map of pCAMBIA1300-YFPc and pCAMBIA1300-YFPn was added in Supplementary S5. All primers were listed in Supplementary Table S1.

### Preparation and infiltration of the *Agrobacterium* culture

The two *Agrobacterium* tumefaciens strains, GV3101 (pSoup-p19) and AGL1, were purchased from Shanghai Weidi Biotechnology Co, Ltd. Vectors carrying the pCAMBIA2300-HopF2-GFP construct were transferred to GV3101 (pSoup-p19) and AGL1 using the heat shock method. The resulting *Agrobacterium* strains were stored at -80 °C in 50% (v/v) glycerol (Sinopharm, China). Frozen *Agrobacterium* stocks were inoculated on solid Luria Broth (LB) plates containing 25 µg/µL rifampicin and 50 µg/µL kanamycin (Sangon, China) at 28 ℃ for 48 h (GV3101(pSoup-p19)) and 72 h (AGL1) respectively, to obtain a single colony.

The next experiment was conducted in four steps. Step 1: At first, a single positive colony of transformed *Agrobacterium* was inoculated into 500 µL of LB liquid medium, which contained 25 µg/µL rifampicin and 50 µg/µL kanamycin. Then, it was incubated at 28 ℃ with continuous shaking at 200 rpm for a duration of 12 to 18 h. Step 2: 200 µL *Agrobacteium* cells in Step 1 were added to 3 mL LB liquid medium (25 µg/µL rifampicin and 50 µg/µL kanamycin) and grown overnight for preculture. Step 3: 200 µL *Agrobacterium* cells in Step2 were transferred to 20 mL LB liquid medium and cultured for approximately 2 h until they reached the logarithmic growth phase (OD_600_ = 0.6 ∼ 0.8). The cells were centrifuged at 6500 rpm for 10 min at room temperature and the supernatant was discarded. The collected cells were then resuspended and diluted to OD_600_ = 0.8 with infiltration solution (1/2MS medium (pH 5.7 ∼ 5.8), 10 mM MgCl_2_ (Sinopharm, China), 10 mM MES monohydrate (Biosharp, China), 200 µM acetosyringone (AS) (Sangon, China)) and ready for infiltration. The resuspended *Agrobacteium* cells were infiltrated into spinach leaves using a 1-mL plastic syringe. Gently infiltrate until the entire leaf was transparent and saturated with *Agrobacteium* cell suspensions. Normally only one injection was required on each side of the leaf vein for individual leaves. Step 4: After drying the surface bacterial solution with paper, the transformed spinach leaves were kept in the dark at 20 ℃ for 12 h and then transferred back to normal conditions (12 h light, 22 °C /10 h dark, 20 °C) for a further 41 ∼ 60 h before detection by microscopy. Transformation efficiency = number of leaves emitting fluorescent signals ⁄ number of leaves injected with *Agrobacteium* cells.

### Fluorescence microscopy

A small piece of leaf was placed onto a glass slide with water drops acting as the mounting medium. GFP signal was visualized using confocal microscope (Olympus FV3000, Japan) with a 488 nm argon excitation laser and 561 nm emission wavelengths. Images were exported as TIFF files to be analyzed. Vector control pCAMBIA2300 without GFP was used for normalization or auto fluorescence correction (Supplemental Fig. S4). The fluorescence intensity of HopF2-GFP at the cell membrane was calculated by the ImageJ software [64, 65].

Each image was converted to a 16-bit format. Denoising and smoothing were accomplished by applying the Gaussian blur function with a sigma radius of 2. The GFP-expressing tissue was separated from the background using the Morphologic segmentation function with the Tolerance of 10. The image was created using the display of catchment basins, and each cell was then individually selected using the wand tool. Binarized images were obtained through creating mask and using Morphological Filters with a radius (in pixels) of 4. ROI was obtained through mask of cell membrane. The mean value is computed from the fluorescence intensity of the selected cell membrane.

### Subcellular localization analysis

The fusion vector pCAMBIA2300-SoMBF1c-GFP and pCAMBIA2300-SoRbohF-GFP were transformed into *A. tumefaciens* GV3101(pSoup-p19) strain, respectively. An empty vector pCAMBIA2300-GFP was used as the negative control. Then, transformed GV3101(pSoup-p19) cells were infiltrated into the G1 when Sp75 seedlings were at the four-leaf stage. The fluorescence signal was observed by laser confocal microscope (Olympus FV3000, Japan) with a 488 nm argon excitation laser and a 561 nm emission wavelengths.

### Bimolecular fluorescent complementary (BiFC) assay

Two constructs pCAMBIA1300-SoCRK10-YFPc and pCAMBIA1300-SoRbohB-YFPn were transformed into *A. tumefaciens* GV3101 (pSoup-p19), respectively. A suspension of *Agrobacteria* was prepared as mentioned above and stand at 28 ℃ for 3 ∼ 5 h before mixing and then injected into spinach leaves for transient expression. Then, spinach plants were placed at 20 °C under dark for 12 h, then transferred to a green-house at 22 °C under 16 h light/8 h dark for 36 ∼ 60 h. GFP fluorescence was observed by laser confocal microscope (Olympus FV3000, Japan) under the excitation of 488 nm lasers and 561 nm emission wavelengths. This experiment was repeated with three replicates.

### Quantitative real time-PCR (qRT-PCR)

The expression levels of the target genes were detected using SYBR Green reagent in a StepOnePlus™ Real-Time PCR system (Thermo Fisher Scientific, USA) and calculated from the cycle threshold based on the 2^−ΔΔCT^ methods. The spinach polyubiquitin gene *SoARF* was used as an internal control. Primer sequences for qPCR are listed in Supplemental Table S1. Experiments were repeated at least three times independently.

### Protein extraction and western blotting

Proteins were extracted from spinach leaves ground in liquid nitrogen. The tissue powder was resuspended in extraction buffer (100 mM Tris-HCl at pH 8.0, 10 mM β-mercaptoethanol (Amresco, USA), 1 mM EDTA (Genview, USA), 0.2 M sugar (Genview, USA) and protease inhibitor (Solarbio, China)). The mixture was vortexed and centrifuged at 20,000 × g for 15 min. The suspensions were boiled in 2 × SDS/PAGE loading buffer and separated by 12% SDS/PAGE. The protein extract was then transferred to a nitrocellulose membrane and HopF2-GFP fusion proteins were visualized on immunoblots using an anti-GFP antibody (Transgen, China).

### GUS staining

The GUS staining protocol was performed as previously described with some modifications [66, 67]. The leaves were immersed in a GUS staining solution consisting of 100 mM sodium phosphate buffer (pH 7.0), 2 mM 5-bromo-4-chloro-3-indolylglucuronide (X-Gluc) (Sangon, China), 0.05 mM K_3_Fe(CN)_6_ (Sinopharm, China), 0.05 mM K_4_Fe(CN)_6_ (Sinopharm, China), and 0.1% (w/v) Triton X-100 (Biodee, China) under vacuum for at least 30 min. They were then incubated at 37 °C for 50 h. To facilitate penetration of the GUS staining solution into the leaf cells, cutting the leaves may be necessary due to the thick waxy layer on the surface of the leaves. Staining was terminated and green coloration was removed using 95% ethanol.

### Electronic supplementary material

Below is the link to the electronic supplementary material.

**Supplementary Material 1**: **Table S1.** The primers used in the study.

**Supplementary Material 2**: **Figure S1**. Quantification the expression of HopF2-GFP by qRT-PCR and western-blot assay. **Figure S2**. The fluorescence intensity of SoRbohF-GFP, SoMBF1c- GFP and GFP control. **Figure S3**. Histochemical assay of GUS expression in transiently expressed spinach leaves. **Figure S4**. Image of vector control pCAMBIA2300 without GFP for normalization or auto fluorescence correction. **Figure S5**. The vector map of linear pCAMBIA1300-YFPc (A) and pCAMBIA1300-YFPn (B).

## Data Availability

All data generated or analyzed during this study are included in this published article and its supplementary information files.
