# Supplementary material for: A high-efficiency transient expression system mediated by Agrobacterium tumefaciens in Spinacia oleracea leaves
Source: Plant Methods. 2024 Jul 2;20:100. doi: 10.1186/s13007-024-01218-y (PMC11220957; doi:10.1186/s13007-024-01218-y)
Supplement: Supplementary file 1 — Supplementary Material 1: Table S1. The primers used in the study. [file 13007_2024_1218_MOESM1_ESM.docx]

**Table S1 The primers used in this study.**

| Primer name | Primer sequence |
| --- | --- |
| SoRbohF-GFP F | TTACAATTAGGATCCTCTAGAATGAGAGGCTACGCGAGGC |
| SoRbohF-GFP R | GTAGTCAGAAGGCCTCCCGGGGAAGTGTTCCTTGTGGAATTCAAA |
| SoMBF1c-GFP F | GGATCCTCTAGAATGCCAACCCGACCAGCTG |
| SoMBF1c-GFP R | AGTCAGAAGGCCTCCCGGGTTTTCCAGATTT |
| SoRBOHB-BIFC F | TGGCGCGCCACTAGTGGATCCATGAGAAAACCAATGGAGATCCA |
| SoRBOHB-BIFC R | CAACTTTTGCTCCATCCCGGGAAAATTCTCCTTGTGGAAATCAAAC |
| SoCRK10-BIFC F | TGGCGCGCCACTAGTGGATCCATGACTAAGGAAATAAGATGGTTTTCAG |
| SoCRK10-BIFC R  qPCR-HopF-GFP F  qPCR- HopF-GFP R | ATCGTATGGGTACATCCCGGGCTACCTAGGTTCCGGTTCAGTGA  CGTAAACGGCCACAAGTTCA  GGCGGACTTGAAGAAGTCGT |
