# Supplementary material for: A high-efficiency transient expression system mediated by Agrobacterium tumefaciens in Spinacia oleracea leaves
Source: Plant Methods. 2024 Jul 2;20:100. doi: 10.1186/s13007-024-01218-y (PMC11220957; doi:10.1186/s13007-024-01218-y)
Supplement: Supplementary file 2 — Supplementary Material 2: Figure S1. Quantification the expression of HopF2-GFP by qRT-PCR and western-blot assay. Figure S2. The fluorescence intensity of SoRbohF-GFP, SoMBF1c- GFP and GFP control. Figure S3. Histochemical assay of GUS expression in transiently expressed spinach leaves. Figure S4. Image of vector control pCAMBIA2300 without GFP for normalization or auto fluorescence correction. Figure S5. The vector map of linear pCAMBIA1300-YFPc (A) and pCAMBIA1300-YFPn (B). [file 13007_2024_1218_MOESM2_ESM.docx]

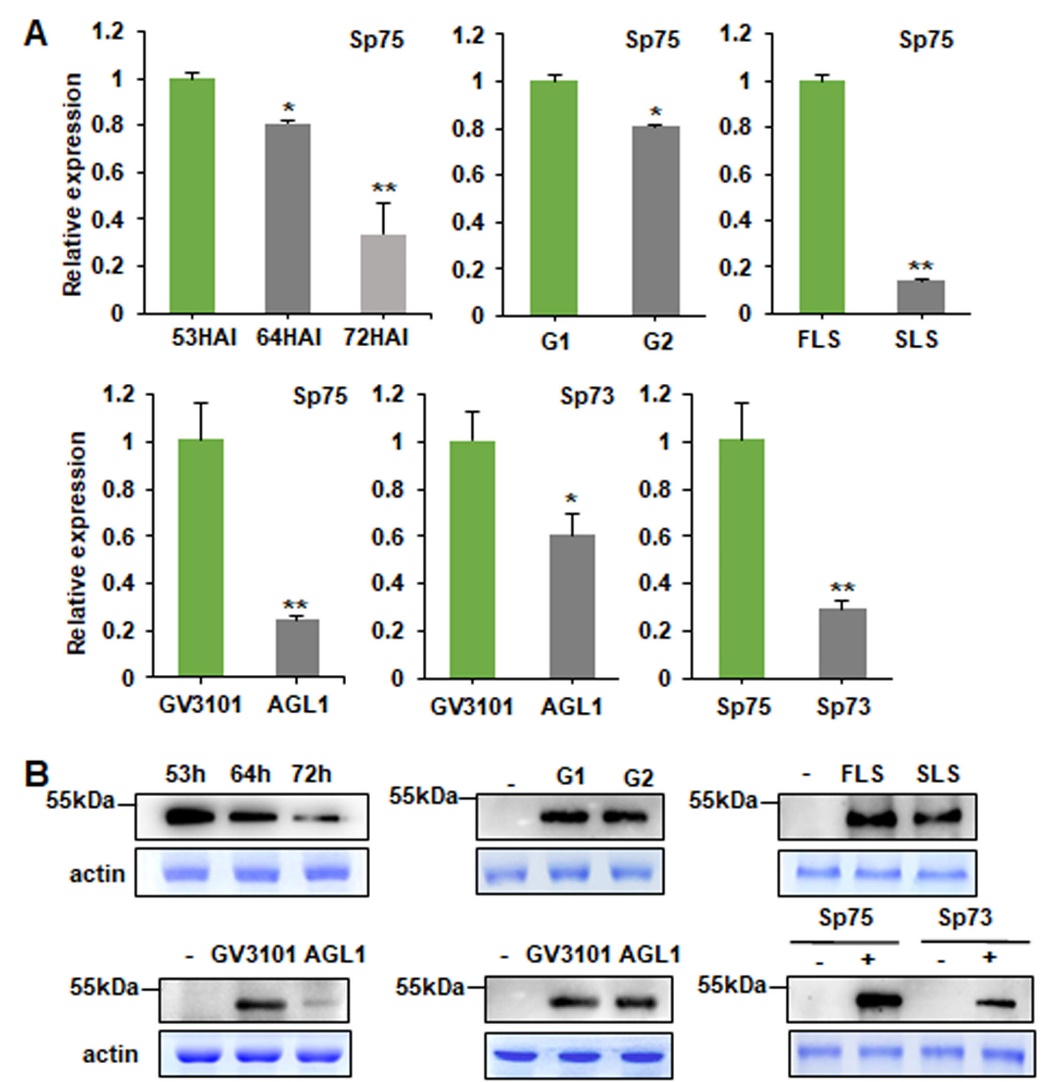


**Figure S1 Quantification the expression of HopF2-GFP by qRT-PCR and western-blot assay.**

**A.** qRT-PCR analysis of *HopF2* gene transcript levels of in G1 of Sp75 infiltrated with GV3101 (pSoup19-p19) at 53 h, 64 h, 72 h after injection (HAI), in G1 and G2 of Sp75 infiltrated with GV3101 (pSoup19-p19), in G1 of Sp75 infiltrated with GV3101 (pSoup19-p19) at four-leaf stage (FLS) and six-leaf stage (SLS), in G1 of Sp75 respectively infiltrated with GV3101 (pSoup19-p19) and AGL1, in G1 of Sp73 respectively infiltrated with GV3101 (pSoup19-p19) and AGL1, and in G1 of Sp75 and Sp73 infiltrated with GV3101 (pSoup19-p19). *SoARF* gene was used as an internal control. Standard deviations were calculated from at least three independent experiments. Data are means of three replicates ± SD, and the asterisks indicate significant differences compared with the first column (*p < 0.05, **p < 0.01, t-test). **B.** Western-blot analysis of the protein levels of HopF2-GFP in (A). Actin was stained with Coomassie Brilliant Blue (CBB) and used as loading control. **-**, proteins extracted from spinach leaves without *Agrobacterium* strain infiltration. **+**, proteins extracted from spinach leaves with GV3101 (pSoup19).


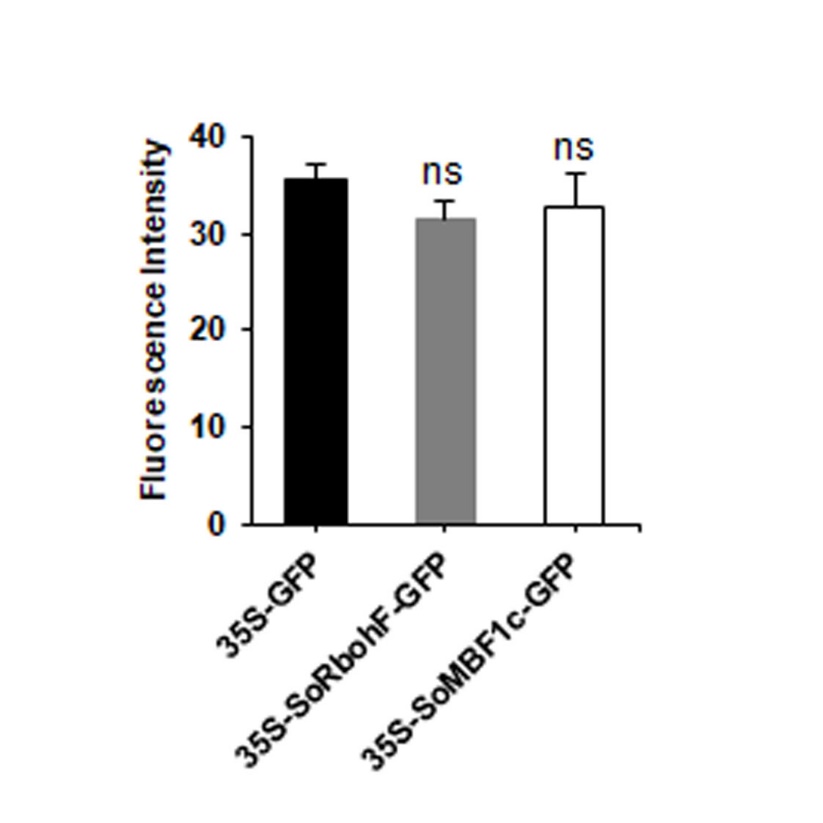
**Figure S2** **The fluorescence intensity of SoRbohF-GFP, SoMBF1c- GFP or GFP control.** *Agrobacterium* strain GV3101 was infiltrated in G1 of spinach Sp75. AU: arbitrary unit. The experiment was repeated three times with similar results. Data presented are mean values ± SD (n ≥ 6). ns: not significance (P > 0.05).


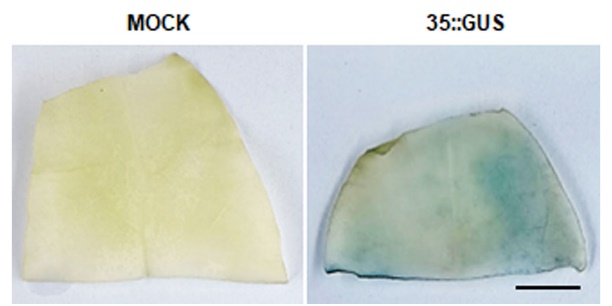


**Figure S3 Histochemical assay of GUS expression in transiently expressed spinach leaves.**

GUS expression was examined in organ cross-sections at 50 hours after infection. Spinach leaves in the same culture condition without *Agrobacterium* infection are indicated (MOCK). Scale bar = 0.5 cm


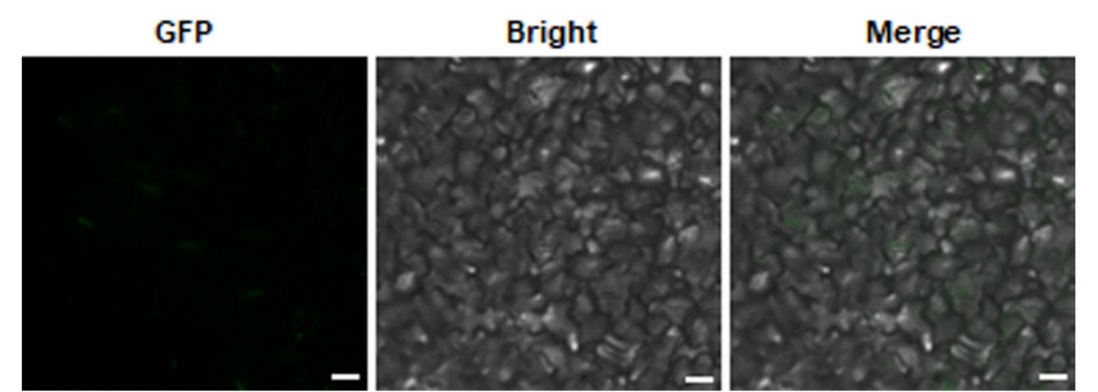


**Figure S4 Image of vector control pCAMBIA2300 without GFP for normalization or auto fluorescence correction.** Scale bar = 50 μm. The experiments were repeated three times with similar results.


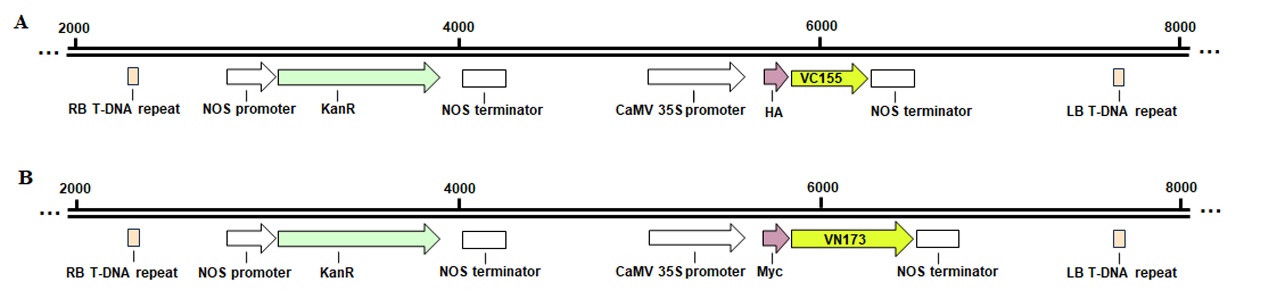


**Figure S5.** **The vector map of linear pCAMBIA1300-YFPc (A) and pCAMBIA1300-YFPn (B).** The left (LB) and right (RB) borders of the T-DNA are shown. HA tag fused-YFPc (VC155) and Myc tag fused-YFPn (VN173) was driven by the *Cauliflower mosaic virus 35S* promoter, respectively. The kanamycin resistance (KanR) gene was under the control of the nopaline synthase (NOS) promoter.
